# Pan-retinal characterisation of Light Responses from Ganglion Cells in the Developing Mouse Retina

**DOI:** 10.1038/srep42330

**Published:** 2017-02-10

**Authors:** Gerrit Hilgen, Sahar Pirmoradian, Daniela Pamplona, Pierre Kornprobst, Bruno Cessac, Matthias H. Hennig, Evelyne Sernagor

**Affiliations:** 1 Institute of Neuroscience, Newcastle University, Newcastle upon Tyne NE2 4HH, UK; 2 Institute for Adaptive and Neural Computation, University of Edinburgh EH8 9AB, Edinburgh, UK; 3 Université Côte d’Azur, Inria, Biovision team, 06902 Sophia Antipolis, France

## Abstract

We have investigated the ontogeny of light-driven responses in mouse retinal ganglion cells (RGCs). Using a large-scale, high-density multielectrode array, we recorded from hundreds to thousands of RGCs simultaneously at pan-retinal level, including dorsal and ventral locations. Responses to different contrasts not only revealed a complex developmental profile for ON, OFF and ON-OFF responses, but also unveiled differences between dorsal and ventral RGC responses. At eye-opening, dorsal RGCs of all types were more responsive to light, perhaps indicating an environmental priority to nest viewing for pre-weaning pups. The developmental profile of ON and OFF responses exhibited antagonistic behaviour, with the strongest ON responses shortly after eye-opening, followed by an increase in the strength of OFF responses later on. Further, we found that with maturation receptive field (RF) center sizes decrease, spike-triggered averaged responses to white noise become stronger, and centers become more circular while maintaining differences between RGC types. We conclude that the maturation of retinal functionality is not spatially homogeneous, likely reflecting ecological requirements that favour earlier maturation of the dorsal retina.

The onset of visual experience in mouse occurs around postnatal day (P) 12, at eye opening. Although the retina cannot experience patterned vision beforehand, it is remarkable that RGCs are already capable of encoding information originating from photoreceptors and transmit it to retinal central targets as soon as eyes open. However, these early light responses are far from mature, and they progressively acquire their adult features while the retina develops^1^,^2^,^3^,^4^. In mouse, RGC dendritic stratification in the ON and OFF layers of the inner plexiform layer matures after eye opening^5^ and light-driven activity guides the refinement of synaptic connectivity^6^,^7^. Consequently, RF sizes^8^,^9^ and complex RF properties such as direction and orientation selectivity^10^,^11^,^12^ keep maturing after the onset of visual experience. Yet, despite ongoing maturation after eye opening, longitudinal studies of RF properties have never been fully documented^3^.

One important often neglected issue is that the retina is not uniformly organised from a functional perspective. Indeed, dorsal, ventral, nasal and temporal domains have evolved to enable optimal encoding of specific features in the visual scene. For example, mouse cones co-express medium wavelength and short wavelength opsins (M-opsin and S-opsin), with a dorsal-to-ventral increasing gradient in S-opsin (and opposite for M-opsin)^13^,^14^,^15^,^16^,^17^,^18^,^19^. These dorso-ventral gradients affect RGC responses in adult animals with respect to their spectral tuning^20^,^21^,^22^, improving encoding of achromatic contrasts^20^,^21^ and providing evolutionary advantages for visual tasks^23^,^24^. The topographical organisation of some RGC subtypes also exhibits dorsal, ventral, nasal, and temporal non-uniformity^25^,^26^,^27^,^28^. However, nothing is known about the developmental consequences of these inhomogeneities.

Here we present a longitudinal study of RGC RF properties in the developing mouse retina from eye opening up to maturity with emphasis on dorso-ventral topographical differences. We recorded simultaneously from hundreds to thousands of RGCs at near pan-retinal level using the high-density large-scale CMOS-based Active Pixel Sensor multielectrode array (APS-MEA) featuring 4096 electrodes (42 μm pitch) arranged in a 64 × 64 configuration, covering an active area of 7.12 mm^2^ 29,30,31, allowing us to discriminate topographical differences in light responses. We classified RGC responses as ON, OFF and ON-OFF by measuring basic firing properties such as latency, peak amplitude and response duration at different contrast levels, despite the fact that under some conditions, RGCs with an ON-OFF morphology reveal responses of one single polarity^32^, and RGCs with an OFF morphology reveal ON responses^33^,^34^. We completed our study by determining the spatio-temporal properties of their RF central areas throughout development using a novel, high resolution stimulus approach.

## Results

### Simultaneous pan-retinal recording from the dorsal and the ventral retina

**Figure 1:**
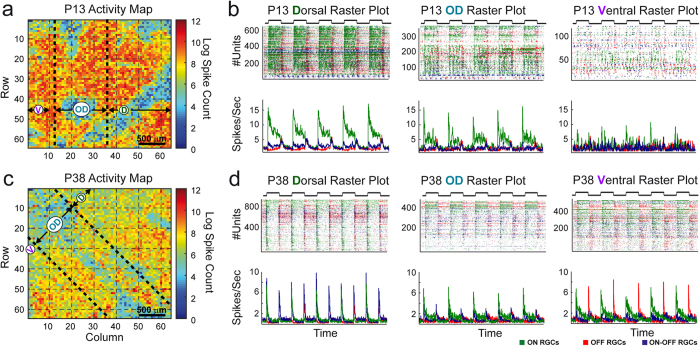
Activity maps and spike raster plots of a P13 and a P38 retina. (**a,c)** The *Log* spike count (full field stimulus experiment) for each channel from a P13 (**a**) and a P38 (**c**) retina is pseudo color-coded and plotted according to electrode coordinates (64 × 64 array). This results in a visualisation of the retina outline and gives an overall estimation of the number of active channels. (**b,d)** Spike raster plots from the same P13 (**b**) and P38 (**d**) experiment used for (**a** and **c**), respectively, but after spike sorting. Each dot is representing a spike in an alternating full field stimulus experiment and dots are color-coded: green = ON RGC responses, red = OFF RGC responses, dark blue = ON-OFF responses and the raster/rate plots are divided into dorsal (**b,d** left), ventral (**b,d** middle) and OD (**b,d** right) locations. The binned (25 ms) average response (Spikes/Sec) of all RGC responses is plotted below the raster plots.

The spatial extent (7.12 mm^2^) of the APS-MEA chip allowed us to record simultaneously from large retinal areas (Fig. 1a and c). The small electrode pitch (42 μm) enables sampling from many individual RGCs from these areas, providing us with an unbiased very large analytical sample size (see Table 1) and helps to reduce the amount of experiments/animals needed. We divided the retina into ventral and dorsal areas according to the retinal orientation. In addition, we assigned a third, transition area around the optic disk (OD). We set the boundaries for the OD area to +/−0.5 mm from the OD according to known cone spatial distribution^19^. The area dorsal to the OD area is richer in short wavelength cones and the area ventral to the OD area is richer in medium wavelength cones^19^. Figure 1 illustrates responses to full field stimuli in a typical immature (P13) and mature (P38) retina. It is immediately obvious that the dorsal area is much more responsive to light than the ventral area at P13 and these differences disappear with maturation (these two examples are rather extreme cases, chosen to emphasize the developmental trend quantified in this study). Supplemental Fig. S1 is showing the quantification of the Peak Amplitudes (*A1, A2*) and the Response Durations (*RD1, RD2*) from these same retinas (detailed p-values in Supplemental Table S14). Figure 1a and c show that it is possible to visualise the boundaries of the retina, to quantify activity levels on individual channels and to delineate various retinal areas on the APS-MEA chip simply by looking at spiking activity. For each channel the log spike count of a full field stimulus experiment (see Methods) from a P13 (Fig. 1a) and an adult (P38, Fig. 1c) retina were pseudo color-coded and plotted according to their position on the 64 × 64 APS-MEA chip. This generates activity maps showing that the outline of the P13 retina is slightly smaller compared to the adult P38 retina and that the spike rate is overall higher in all channels in that younger retina. Figure 1b and d illustrate responses to these same full-field stimuli in both retinas following spike sorting and RGC response classification (see Methods), yielding spike rasters and histograms for dorsal (D), ventral (V) and around the OD (OD) located ON (green), OFF (red) and ON-OFF (dark blue) RGC responses. The responses of P13 ON cells to the alternating full field stimulus were much stronger and more sustained compared to the responses of OFF and ON-OFF RGC in the same retina (Fig. 1b) and to all responses in the P38 retina (Fig. 1d). But OFF and ON-OFF cell responses became temporally more precise at P38 than at P13. Interestingly, the P13 ON cell responses were stronger in the dorsal than in the ventral and OD part of the retina (Fig. 1b). These regional differences become less predominant with retinal maturation.

**Figure 2:**
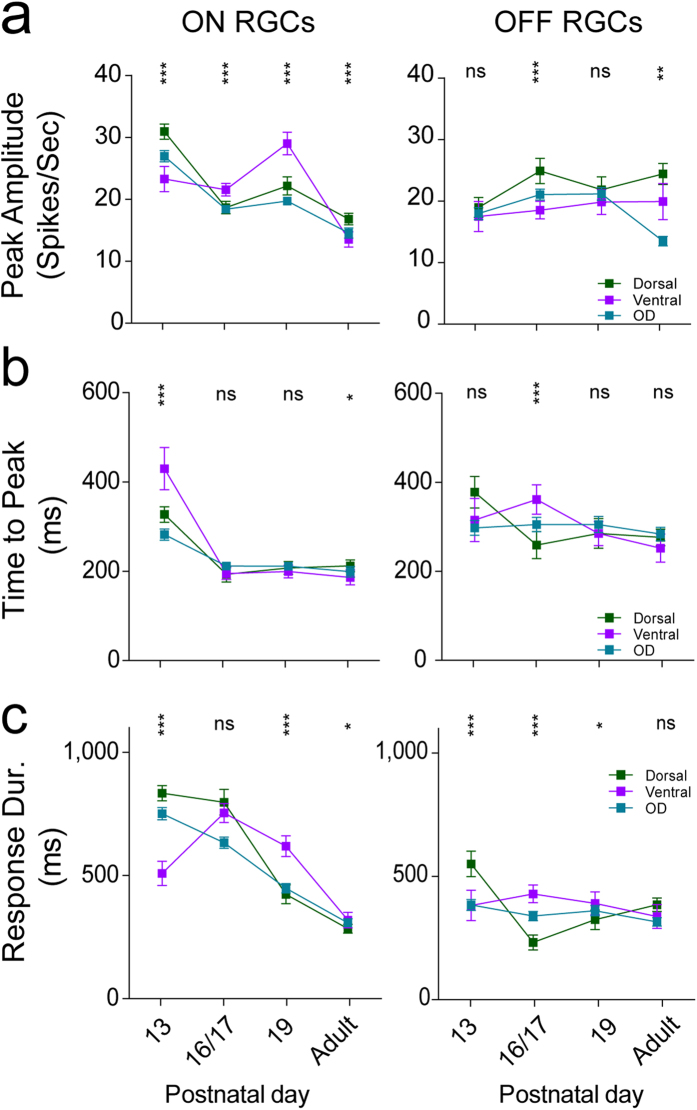
Properties of different RGC response types from P13 to adult. (**a,b,c)** Respectively illustrate peak amplitude (*A1, A2*), time to peak (*T2P1, T2P2*) and response duration (*RD1, RD2*) for dorsal (green), ventral (purple) and OD (turquoise) ON (left) and OFF (right) responses for each age group (mean values with 95% confidence interval, n: see Table 1). Significance asterisks are only displayed for dorsal and ventral comparison: *p < 0.05; **p < 0.01; ***p < 0.001; ns = not significant. Detailed p-values can be found in Supplemental Table S9.

**Figure 3:**
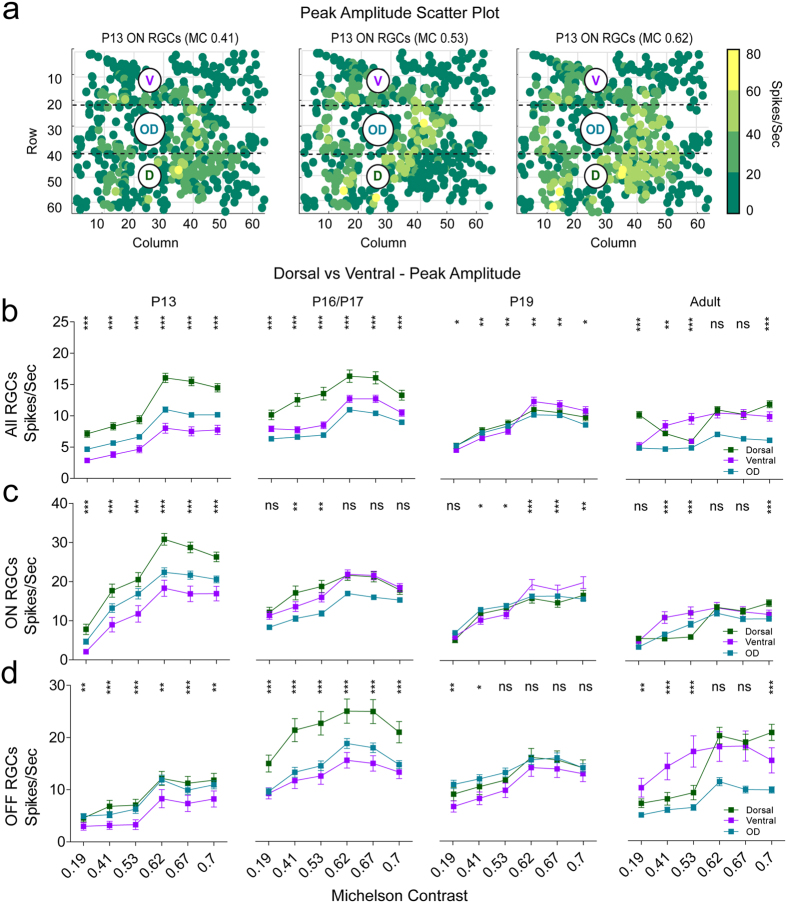
Dorsal-ventral gradient of peak amplitudes to different contrasts after eye-opening. (**a)** ON peak amplitudes (*A1* in Spikes/Sec) for three different full field contrasts (0.41, 0.53, 0.62) from a single P13 retina are plotted in pseudo colours according to firing strength and electrode position. For visualisation, the individual x, y electrode position for the peak values was slightly randomly shifted (+/−0.25) because after spike sorting multiple RGC units are assigned to the same electrode position. (**b–e)** Mean peak response amplitudes to different full field Michelson contrasts (0.19, 0.41, 0.53, 0.62, 0.67) for all (**b**), ON (**c**) and OFF (**d**) response types for all age groups (ascending from left to right) with respect to their dorsal (green). ventral (purple) and OD (turquoise) location. All plot conventions are like for Fig. 3. Detailed p-values can be found in Supplemental Table S10.

**Figure 4:**
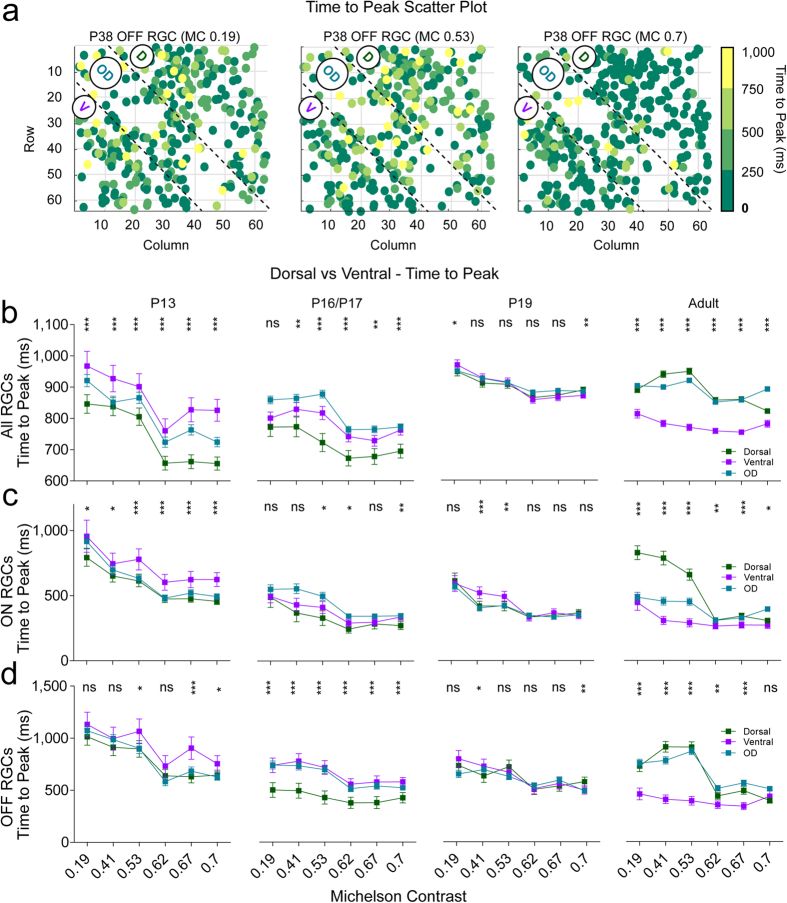
Dorsal-ventral differences for the time to peak values at lower contrast in the adult retina. (**a)** OFF RGC response color-coded times to peak (*T2P2* in ms) from a single P38 at three different full field contrasts (0.19, 0.53 and 0.7) plotted according to their electrode position. (**b–e)** Mean times to peak plotted for the different full field Michelson contrasts (x axis, 0.19, 0.41, 0.53, 0.62, 0.67) for all (**b**), ON (**c**) and OFF (**d**) responses and all age groups. All plot conventions are like for Fig. 3. Detailed p-values can be found in Supplemental Table S11.

**Figure 5:**
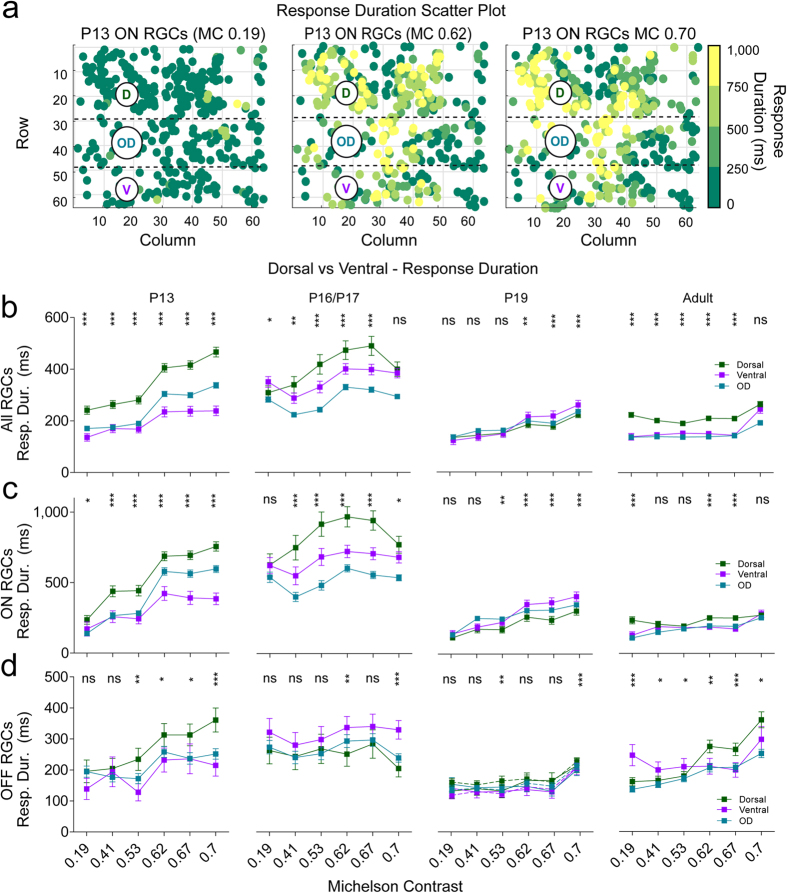
Responses to light are more sustained for all dorsal RGCs than for their ventral counterparts. (**a**) The ON RGC response duration times (*RD1* in ms) from a single P13 retina of three different full field contrasts (0.19, 0.62, 0.7) were pseudo colour-coded plotted according to their electrode position. (**b–e)** The mean response duration times to different full field Michelson contrasts (x axis, 0.19, 0.41, 0.53, 0.62 and 0.67) for all (**b**), ON (**c**) and OFF (**d**) RGC responses and all age groups. All plot conventions are like for Fig. 3. Detailed p-values can be found in Supplemental Table S12.

This simple pan-retinal visualisation demonstrates that the development of basic firing properties varies not only for different RGC response types, but even for responses we found in dorsal versus ventral ON and OFF RGCs. Next, we quantified these stimulus-driven responses for different RGC response types at different ages (Fig. 2), and we examined how responses change when the full-field stimulus was presented with different Michelson contrasts (Figs 3, 4 and 5).

### Ontogeny of dorsal and ventral light response features for different RGC response types

In Fig. 2 we present results for ON and OFF RGC responses, but the analyses were also carried out on ON-OFF RGCs and can be found in Supplemental Fig. S2. Further, to avoid overcrowding the plots, the asterisks in the figures refer only to statistically significant differences between the dorsal and ventral data.

Full field stimuli were presented to retinas of different ages, and post-stimulus time histograms (PSTH) were generated for every RGC. The results were classified into 4 age groups: P13 (4 retinas, 4,183 RGCs in total), P16/P17 (2 P16 and 2 P17 retinas, 4,404 RGCs), P19 (5 retinas, 7,094 RGCs) and adult (2 P29 and 2 P38 retinas, 5,811 RGCs) and further divided into the major groups ON, OFF and ON-OFF RGC responses (see Methods and Discussion) and additionally into dorsal, ventral and OD-located RGC responses (Table 1). For the results in Figs 2–5 we are referring to these groups. From individual PSTHs, we extracted the peak amplitude, corrected for baseline firing (see Methods), time to peak and response duration for light onset and offset. Figure 2a summarises results for ON (left) and OFF (right) RGC responses at all ages (x-axis), illustrating the dorsal (green), ventral (purple) and OD (turquoise) mean peak amplitudes *A1* (ON cells) and *A2* (OFF cells). In line with the example in Fig. 1b, dorsal P13 ON cells were significantly more active than ventral P13 ON (Fig. 2a, left) and all OFF cells in all areas. There is also a progressive decrease in *A1* from P13 to adulthood for ON cells, whereas *A2* increases with age for ventral and dorsal OFF cells but not for OD OFF cells (Fig. 2a, right). The peak observed for ventral P19 ON cells is surprising. We verified whether this observation could be an outlier, but except for 1 retina (#1 in Supplemental Fig. S4) that behaviour was consistent upon inspection of baseline firing and individual spike rasters for all five retinas at that age (see Supplemental Fig. S4), hence it is not an artefact. Both, ON and OFF peak responses were significantly different in adult retinas.

How fast these responses to full field stimuli peak at different ages was evaluated by measuring the time from stimulus on- or offset to the peak amplitude (*T2P1* and *T2P2,* respectively). We found that *T2P1* in ON cells and *T2P2* in OFF cells progressively decrease (less marked in OFF cells) from P13 to adulthood. OFF cells exhibited overall slower time to peak values than ON cells with maximum values at P13 for dorsal, P16 for ventral and P19 for OD cells (Fig. 2b, right).

We also measured the response duration for ON and OFF cells (*RD1* and *RD2*, respectively). Dorsal ON and OFF responses are significantly more sustained at P13 (Fig. 2c, left and right). Ventral ON responses steadily increase up to P19 and then they drop down to a minimum. Subsequently, dorsal ON cells become gradually more transient with development (Fig. 2c, left), from P16/17 onwards their *RD1* values became lower than ventral *RD1.* Interestingly, adult ventral ON responses are more sustained than dorsal ON responses whereas ON-OFF responses show the opposite effect (Fig. S2). OFF cells become moderately more sustained between P16 and adulthood, eventually producing adult responses of similar duration like ON cells (Fig. 2c, left and right). The developmental patterns of ON and OFF OD cells were almost similar to ON dorsal cells and OFF ventral cells respectively.

Taken together, dorsal light responses are more prominent after eye-opening and the peak amplitudes and response durations of ON and OFF cells showed an antagonistic behaviour from eye opening to maturity.

### Ventral and dorsal difference–peak amplitude

Next, we investigated how these developmental changes vary with the stimulus contrast. Using the same age groups as above, we recorded full field responses with different Michelson contrasts (MC, see Methods). Figure 3a illustrates the firing peak amplitudes for all ON cells (*A1*) from a P13 retina for three different full field contrasts (0.41, 0.53, and 0.62) plotted with respect to their electrode position on the array. Overall, the dorsal side clearly shows stronger activity (yellow), especially for MC 0.62. To establish whether this dorsal-ventral trend is present in all retinas of the same age group and/or between the age groups, we calculated *A1* and A2 for all full field contrasts for all RGC response types (Fig. 3b) including the ON, OFF and ON-OFF responses. At P13, all dorsal RGC responses exhibited significantly higher peak firing rates than ventral cells at most contrast levels (Fig. 3b–d left). The highest values and most pronounced differences between dorsal and ventral cells were observed at 0.62 Michelson contrast rather than at the highest contrast (MC 0.7). At P16/17, dorsal OFF RGCs were also significantly more active than their ventral counterparts (Fig. 3d middle), whereas there were no significant differences between dorsal and ventral ON cells (Fig. 3c middle). At P19 no dorsal and ventral differences were found except that ventral ON cells become significantly more active than their dorsal counterparts for the three strongest contrast levels (Fig. 3c middle), in line with the results in Fig. 3a. For adult retinas, we found stronger ventral responses at lower contrast levels, an effect that switched to dorsal at the highest contrast level (Fig. 3c and d right). Taken together, dorsal units are strikingly more active after eye-opening, an effect that disappears in older retinas.

### Ventral and dorsal difference–time to peak

Figure 4a shows *T2P2* values for all OFF cells from a mature P38 retina for three different full field contrasts (MC 0.19, 0.53, 0.7). Here, dorsal responses appear more sluggish for lower contrasts. Similar trends were observed for all adult RGC response types (Fig. 4b–d right), with responses in dorsal RGCs s showing significantly longer times to peak than ventral cells, a phenomenon especially prominent at lower contrasts. However, at P13 all ventral ON RGCs had more sluggish responses than dorsal cells at the highest MC levels (Fig. 4c, left), while this difference between dorsal and ventral responses was also observed at lower contrast in OFF P16/P17 RGCs. There were no differences between dorsal and ventral ON P16/P17 and ON and OFF P19 RGC responses (Fig. 4c, middle). In summary, there is a negative developmental shift of the response time to peak for all RGCs, with the most sluggish responses found in ventral RGCs s after eye-opening and slower responses in dorsal units in adults.

### Ventral and dorsal difference–response duration

Figure 5a illustrates response durations for all ON cells in a P13 retina for three different contrast levels (MC 0.19, 0.62, 0.7), with longer responses developing at higher contrasts in the dorsal part of the retina. At P13 dorsal RGC responses were more prolonged at most contrast levels (Fig. 5b–d left), and there is a similar trend for ON cells at P16/17 (Fig. 5c, middle). As for the other age groups, differences between dorsal and ventral cells became virtually non-existent, except for small differences at higher contrast levels (e.g. longer ventral OFF responses at MC 0.7 at P16/17 and P19; longer dorsal ON and OFF responses at higher contrast levels in adult retinas).

In summary, there are significant differences between the dorsal and ventral properties (peak firing, time to peak and response duration) for different RGC response types from eye-opening to maturity. Shortly after eye opening, responses in dorsal RGCs are much stronger and more sustained to full field flashes than in ventral cells. These dorsal-to-ventral differences fade as development progresses, and interestingly, in many cases the gradient even reverses to ventral-to-dorsal.

### Maturation of RGC RF centers

**Figure 6:**
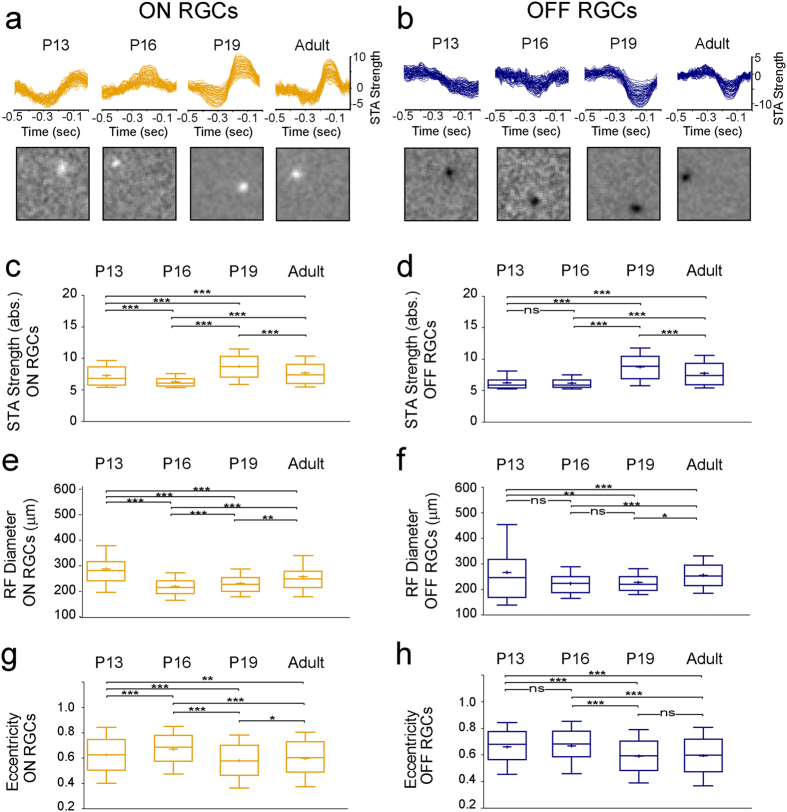
Shifted White Noise used to study the development of RGC RF central areas. (**a,b)** STA signal strength (top) and spatial profiles (bottom) for selected example RF central areas from ON (**a**) and OFF (**c**) RGCs from all 4 age groups (see below). (**c–h)** Box plots (whiskers: 10–90 percentile, mean indicated by + symbol) of ON (yellow boxes; P13-N = 2 retinas, n = 449 cells; P16-N = 2, n = 425; P19-N = 2, n = 926; adult-N = 2; n = 698) and OFF (blue boxes; P13-N = 2, n = 309; P16-N = 2, n = 239; P19-N = 2, n = 794; adult-N = 2, n = 514) RGC responses from all retinal areas for STA signal strength (**c,d**), RF diameters (**e,f**), and RF eccentricity (**g,h**). Significance: *p < 0.05; **p < 0.01; ***p < 0.001; ns = not significant. Detailed p-values can be found in Supplemental Table S13.

We used a novel checkerboard stimulus, shifted white noise (SWN, see Methods), to characterise the maturation of ON and OFF RGC RF centers from eye-opening to maturity. As shown in Pamplona *et al*.^35^, shifted white noise stimulus considerably improves the spatio-temporal resolution of the receptive field computed via spike triggered average (STA). It allows much more accurate receptive field characterisation, which is important to thoroughly describe the variability in receptive field measurements across development. STA is the mean stimulus that precedes a spike and the cell type is inferred from the STA polarity for the phase closest to the spike time 0 (negative for OFF and positive for ON RGC responses). Figure 6 shows the temporal STA signal strength (see Methods) and the corresponding spatial profile for selected ON (yellow, Fig. 6a) and OFF (blue, Fig. 6b) example cells from all used age groups. The most striking developmental change we observed is that both negative and positive STA peak signal strength values are much weaker at P13-16 than later during development. Quantifying the STA strength for all ON (Fig. 6c) and OFF (Fig. 6d) cells shows that it significantly increases during development, with the main change occurring between P16-19. We did observe significant but inconsistent differences in STA strength between dorsal and ventral ON or OFF cells at most ages (see Supplemental Fig. S5). Overall, we found that responses were significantly stronger from P19 onwards.

We next used the STA to measure RF diameters (see Methods) and quantify their developmental changes from eye-opening to maturity. Figure 6e and f show that RF diameters are largest at P13 both in ON (e) and OFF (f) RGCs. dropping down to a minimum at P16, followed by a marginal, non-significant increase at P19 and then a more significant increase from P19 to adulthood. Since we observed significant differences in firing properties between dorsal and ventral RGC response types, we also looked at RF sizes separately for these same two groups, but we found no consistent differences (see Supplemental Fig. S5).

Finally, we quantified the shape of RFs by measuring their eccentricity (see Methods). As shown in Fig. 6g and h, RF eccentricity for both ON and OFF RGC response types increases from P13 to P16, then drops down to a minimum at P19 and increases again in mature cells; that is, the shape of RFs becomes closer to circular after P19. Again, we did not find any consistent differences between dorsal and ventral eccentricities (see Supplemental Fig. S5).

## Discussion

In this comprehensive study we have shown that the basic features characterising RGC light responses have a developmental profile that depends both on cell type and retinal location. Furthermore, these properties do not develop synchronously in the dorsal and ventral retina. Finally, using a novel stimulus, we have been able to reliably characterise RGC receptive fields across development, which is difficult in young retinas where light responses to conventional white noise stimuli are particularly weak.

A recent study showed that at least 30 RGC functional groups exist in the mouse retina^36^. Such detailed classification is however not feasible in immature retinas. Indeed, the responses to light in a P13 retina are very immature shortly after eye opening (around P12) and it is literally impossible to cluster beyond basic types (ON, OFF and ON-OFF) and direction/orientation selective cells consistently through retina development^10^,^11^,^12^,^37^. An additional problem is that temporal parameters used to establish sustained or transient criteria are not robust enough in maturing RGCs, as we show in our study (see also Supplemental Fig. S6). We are aware that our pooling approach into these three major groups could possibly mask additional differences between cells of the same polarity found in the dorso-ventral axis, but any other approach would go beyond the aim of this study or is not really feasible. We classify our RGC responses into ON, OFF and ON-OFF groups using Bias Index values that best separate the overall response polarity, and also ensure that changing the threshold values for classifying cells into these groups has literally no effect on the response behaviour (see Supplemental Fig. S7).

We found that ON responses to light are stronger than other response types just after eye opening. As development progresses, these responses become weaker while OFF responses gain strength (antagonistic to ON cells). The relatively high levels of spontaneous activity (residual of spontaneous waves^30^) could not explain these results because peak amplitudes were normalised to baseline activity. Moreover, if spontaneous activity was indeed affecting our measurements, it would equally affect all RGC responses, and not just ON responses (see Supplemental Fig. S8).

The different maturation time course of ON and OFF peak firing responses may stem from differences in maturation of their surround. The developmental time course of inhibitory neurotransmitter receptor expression is different for particular cell types^38^,^39^. A good candidate to explain our results is the GABA_c_ receptor, expressed presynaptically on bipolar cell axon terminals^40^. Retinal GABA_C_ receptor knockout results in stronger and more prolonged spiking activity^41^, similar to our observations in young ON RGC responses. Further, GABA_C_-receptor mediated inhibition affects the regulation of glutamatergic synaptic transmission at the ON but not at the OFF bipolar-RGC synapse^42^. However, the nature of light-evoked GABAergic inputs onto developing mouse bipolar cells, and how it differs for different RGC types remains to be determined (but see Discussion in^43^). An alternative explanation is that the expression of ionotropic glutamate receptors (AMPA/Kainate and NMDA) differs in ON and OFF RGCs. The NR2A subunit of the NMDA receptor is predominantly found at OFF synapses, while NR2B subunits are preferentially located at ON synapses in rat RGCs^44^. However, Stafford *et al*.^45^ found no evidence for a differential localisation of the NR2B subunits at ON and OFF synapses in direction selective RGCs until P28, but no data is yet available for non-direction selective RGCs. Therefore further studies which combine double labelling of specific cell types and various glutamate receptors through development would be required.

The durations of ON and OFF responses (Fig. 2c) in our study showed a similar antagonistic behaviour as the peak amplitudes (Fig. 2a), but with a much more prominent decrease for ON than for OFF responses, and a massive drop during the fourth postnatal week (Fig. 2c). In addition to the aforementioned points for the GABAc receptor hypothesis, GABA_C_ receptors consist of ρ subunits and the ρ1 subunit expression peaks around P9 (start P6) and ρ2, around P15 (start P9)^38^. The difference in the timing of peak expression of these subunits may explain the differences we observe between ON and OFF response types. Response latencies did not exhibit conspicuous differences between different RGC response types from eye opening to adulthood and the changes we observed most likely reflect ongoing activity-mediated refinement of the bipolar-RGC synapse^2^,^5^,^7^,^43^,^46^,^47^.

In mouse, S-opsins are co-expressed with M-opsins in cone photoreceptors and there are more S-opsins expressed in the ventral retina. S-opsin exhibit peak excitation around 360 nm^22^,^48^, which is in the UV spectrum. To stimulate retinas in our study, we have used a broad spectrum white light composed of red, green and blue LED lights (~420–660 nm) but not UV light. Therefore it is highly unlikely that we were able to stimulate S-opsin properly. Under these conditions, dorsal RGC responses have an advantage because our white light source is biased towards activation of M-opsin, which is more prevalent in the dorsal retina. Yet, our results do not demonstrate a general increase of response strength in the dorsal side *per se,* but reveal a differential dorsal/ventral development pattern for different RGC response types through maturation. Even though a bias in M-opsin activation could potentially explain the stronger responses we recorded at P13, it cannot be responsible for the results we found at later developmental stages, including a switch to stronger ventral responses at low contrast levels in the adult retina. S- and M-opsins are expressed before eye-opening, respectively from ~P1 and ~P8^14^,^15^. Therefore we can reasonably assume that both S- and M-opsin gradients are already fully established shortly after eye opening, when we sampled the earliest light responses, and differences in opsin expression are unlikely to explain our findings. Further, the light intensity (mean luminance 11 cd/m^2^) in our experiments was set to co-activate rods and cones. Therefore rod-mediated (by rhodopsin in the visible spectrum) responses are not saturated in our recordings^49^, and they probably reflect a large proportion of our recordings because rods outnumber cones by 35:1 in the mouse retina^50^. However, early responses (at P13) may be biased towards cone-mediated responses. Indeed, cones mature earlier than rods. They are generated prenatally whilst rod generation spans a longer period, ranging from E12 to P10. Therefore, we expect rod responses to be weaker shortly after eye opening^51^. On the other hand, rods are evenly distributed across the entire retina, so we do not expect dorso-ventral differences. Therefore, the dorso-ventral differences we see at P13 must stem from differences in cone function. Stronger responses in the dorsal retina at the onset of visual experience is not an unreasonable possibility from an ecological and evolutionary point of view because at that age, pups are still gathered in the nest, near the mother, with no ecological need to look skywards, but they rather concentrate on the nest scenery, using dorsal retinal vision. In the ensuing week, retinal circuits keep developing and refining, leading to maturation of the ventral circuitry as well. On a further note we observed occasionally that the OD responses are lower than in the other areas. We do not have an explanation for this phenomenon but it might be related to the fact that this area is densely packed with axonal bundles. It is difficult to isolate these axonal responses and they may arise from distal RGCs which have their somata localised in the ventral or in the dorsal retina.

Presenting white noise checkerboard images and performing a post hoc reverse correlation is now a standard approach to estimate RGC RF centers^52^,^53^. However, the technique is challenging in very young retinas because of high levels of spontaneous activity and the weak sensitivity to light in young RGCs immediately after eye opening. The problem can be alleviated by increasing the number of trials and/or the pixel size without compromising the minimum resolution for reliable RF estimation^8^,^9^,^54^. Here we applied a novel approach, the SWN, which uses large checkerboard pixels (hence evoking strong responses) randomly shifted in space and time by a fraction of the pixel size, yielding better resolution. SWN images are uncorrelated across time, and the bias introduced by the correlation in space due to finite pixel size is negligible. Using this approach, we have shown that STA signal strength defined by the z-score (see Methods) peaks at P19 for ON and OFF RGC response types which is in line with Cantrell *et al*.^8^, who showed a signal strength peak at P18. The significantly higher signal strength in adult ventral RGCs is consistent for ON and OFF responses but antagonistic to our full field results (and also not consistent for the other ages). Further studies are thus needed to investigate the spatial response inhomogeneities in adult retinas. The main developmental increase in STA signal strength peak correlates well with the end of the programmed cell death period for bipolar cells and outer rods^55^, suggesting that the synaptic connectivity required for mature RF center responses is complete at that time, and only minor synaptic refinements occur later on, resulting in further RF expansion. We found that immature (P13) RF diameters in RGCs of the ON response type are significantly larger than later in development, while for cells of the OFF type, RFs are almost similar in size at P13 and in adults, with a temporary drop during the third postnatal week, but the variance at P13 is much higher, which makes it difficult for statistical comparison with the other ages. Previous studies which used conventional checkerboard images present a different developmental picture. In line with our study, Koehler *et al*.^9^ showed that ON and OFF RFs are smaller in adults than immediately after eye-opening, whereas Cantrell *et al*.^8^ stated that OFF, but not ON RFs expand between P15-18 and that there is a significant difference for ON, but not for OFF cells between P18-25. This demonstrates that different checker board approaches and the use of pre- and post-processing steps like RF fitting parameters, RGC selection and RGC classification can yield different results, as elaborated in another study^35^. Additional factors such as mean luminance, adaptational state and recording length may account for these differences, and results should therefore be interpreted with caution. From our measurements of RF eccentricity, we found that RFs become more circular between P16-19. Interestingly this goes in line with a previous study in turtle where anisotropic properties of spatiotemporal RFs were shown to decrease with maturation^3^. These changes are probably caused by the same factors responsible for developmental changes in STA signal strength. We found marginal differences between dorsal and ventral RF properties but since our classification approach is basic (due to difficulties in classifying very young cells, see above), individual RGC response type differences may be masked and further studies are needed to establish more subtle differences.

In summary, using a large-scale, high-density multielectrode array, we have investigated the ontogeny of light responses in RGCs in the developing mouse retina. We were able to record from hundreds to thousands of RGCs simultaneously at pan-retinal level, and found that the refinement of RF properties strikingly differs between the dorsal and the ventral retina, regardless of the response types in individual RGCs. These findings suggest that retinal functionality is not spatially uniform and that there might be an ecological advantage to favouring the development of dorsal light responses before the rest of the retina reaches functional maturity.

## Materials and Methods

### Retina Preparation

All experimental procedures were approved by the ethics committee at Newcastle University and carried out in accordance with the guidelines of the UK Home Office, under control of the Animals (Scientific Procedures) Act 1986. Male and female wild-type mice (C57bl/6), housed under a 12 hour light-dark cycle and aged between postnatal days (P) 13-63 were used for the experiments. Mice were dark-adapted overnight and killed by cervical dislocation. Eyes were enucleated, and following removal of the cornea, lens, and vitreous body, they were placed in artificial cerebrospinal fluid (aCSF) containing the following (in mM): 118 NaCl, 25 NaHCO_3_, 1 NaH_2_ PO_4_, 3 KCl, 1 MgCl_2_, 2 CaCl_2_, 10 glucose, and 0.5 l-Glutamine, equilibrated with 95% O_2_ and 5% CO_2_. The ventral and dorsal orientation was marked after enucleation and confirmed by using vascular landmarks in the retina^56^. The retina was isolated from the eye cup and flattened for MEA recordings and the ventral-dorsal, nasal-temporal orientation was noted down. All procedures were performed in dim red light and the room was maintained in darkness throughout the experiment.

### APS-MEA recordings

The isolated retina was placed, RGC layer facing down, onto the APS-MEA and flattened by placing a small piece of translucent polyester membrane filter (Sterlitech Corp., Kent, WA, USA) on the retina followed by a home-made anchor. Similarly as described elsewhere^30^,^31^, throughout recording, retinas were maintained at 33 °C using an in-line heater (Warner Instruments LLC, Hamden, CT, USA) and continuously perfused using a peristaltic pump (~1 ml min^−1^). Pan-retinal recordings were performed on the BioCam4096 platform with BioChips 4096S+ (3Brain GmbH, Lanquart, Switzerland), integrating 4096 square microelectrodes (21 × 21 *μ*m, pitch 42 *μ*m) on an active area of 2.67 × 2.67 mm. The platform records at a sampling rate of 7.1 kHz/electrode when using the full 64 × 64 array and recordings were stored at 12 bits resolution per channel with a 8 kHz low-pass filter/0.8 Khz high-pass filter using 3Brain’s BrainWave software application. Data management and analysis for large-scale, high-density APS-MEA recording is particular difficult (as reviewed in ref. 57). To reliably extract spikes from the raw traces we used a quantile-based event detection^30^,^58^ and single-unit spikes were sorted using the T-Distribution Expectation-Maximisation algorithm in Offline Sorter (Plexon Inc, Dallas, USA). Sorted units that exhibited at least >0.1 spikes/sec on average over the entire recording session were then verified by visual inspection of the detected clusters in the 2/3D principal component feature space, the calculated cluster interspike intervals with respect to the refractory period and the shape of the spike waveforms in the Offline Sorter GUI. Due to the high density of the electrodes, the same units were sometimes detected on multiple neighbouring channels. These redundant units were removed by comparing coincident spikes between neighbouring units. Briefly, for each unit, spikes occurring within +−2 frames (1 Frame = 1/7.06 ms) were detected in all units on the four closest electrodes and marked. This was done for all units, and units with more than 5% coincident spikes were iteratively removed such that for each coincident group only the one with the largest spike count was retained. On average we finally record from multiple hundreds to thousands individual RGCs in one experiment.

### Light stimuli

Light stimuli were projected onto the retina as described previously^31^,^57^ by exploiting the temporal and spatial resolution of the experimental platform and were attenuated using neutral density filters to high mesopic light levels (mean luminance 11 cd/m^2^).

**Figure 7:**
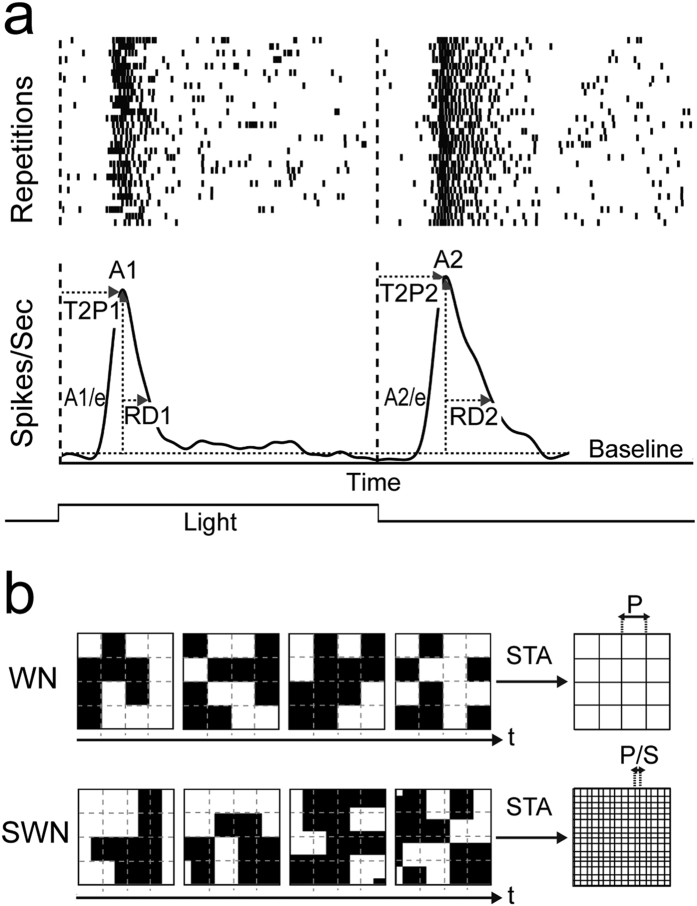
Response parameter calculations and shifted white noise paradigm. (**a)** Top row is showing a spike raster of a full field stimulus (30 trials, 2 sec on, 2 sec off) where each dot is representing a spike, bottom row shows the averaged trials (see methods, converted to Spikes/Sec). Baseline firing rate was estimated by looking at each unit’s activity (Spikes/Sec) before stimuli were presented (>30 sec). *A1* (Spikes/Sec) is the first maximum peak (measured from each unit’s baseline) after stimulus onset and *A2* the first maximum peak after stimulus offset. *T2P1* (in ms) is the time from stimulus onset to *A1, T2P2* the time from stimulus offset, respectively. *RD1* (in ms) is the time, so-called response duration, after *A1* where the response is still above *A1/e. RD2* is the response duration after *A2* to *A2/e*. (**b)** Top is showing the standard white noise paradigm with static located pixels and the resulting resolution (*P*) of the spatial STA profile. Bottom is showing the shifted white noise paradigm obtained by shifting randomly checkerboard images at each time step using *S* possible values of shift in *x* and *y* (see Eq. (1)). This results in a much finer resolution (*P/S*) for the spatial STA profile.

A full field stimulus that switched from light to dark (0.5 Hz, 30 repetitions) was used to define peak response latency, duration and relative amplitude of ON and OFF responses (see Fig. 7a). The luminance contrast for this stimulus measured as the Michelson contrast was defined as (*I*_max_ − *I*_min_)/(*I*_max_ + *I*_min_) where *I*_max_ and *I*_min_ are respectively the maximum and minimum luminance and had a maximal value of 0.70. We also used full field stimuli with a series of increasing Michelson contrasts (0.19, 0.41, 0.53, 0.62, and 0.67). We estimated each unit’s instantaneous firing rate for the different full field intensities by convolving its spike train with a Gaussian kernel smoothing function (standard deviation = 25ms). We then averaged the trials (Fig. 7a) and extracted several features like the amplitude of ON and OFF responses (*A1, A2*), the time from stimulus onset or offset to peak of these response (*T2P1, T2P2* respectively) and the response duration (*RD1, RD2*). Statistical significance was evaluated using an unpaired t-test (two-tailed) (Prism, GraphPad, CA). To classify RGC responses according to their main polarity, we measured the relative amplitude of ON and OFF responses and calculated the *Bias Index (BI*) defined as (*A1* − *A2*)/(*A1* + *A2*) (Carcieri *et al*.^59^). We used the *BI* to classify the cells into OFF (*BI* −1 to −0.33), ON-OFF (*BI* −0.33 to 0.33) and ON cells (*BI* 0.33 to 1).

### Shifted White Noise

Checkerboard stimuli are routinely used to measure RF areas^52^,^53^. Finer resolution can be achieved using smaller unitary checkerboard pixels, but at the same time very small checkerboard pixels may not be able to elicit reliable and repeatable responses, if at all, necessitating to reach a trade-off. Here we use an improved checkerboard stimulus, so-called shifted white noise (SWN), where checkerboard pixels are shifted randomly in space at fixed time steps^35^. With this novel approach, the checkerboard pixel size is large enough to reliably evoke significant responses, but at the same time, the RF resolution can be very fine, given by the shift size (Fig. 7b). The SWN at position *x, y* and time *t* is defined as:


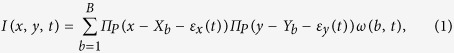


which can be explained as follows. Each image at a given time *t* is composed of *B* checkerboard blocks with a fixed size *P*, the area of each block is defined by a rectangular function *Π*_*P*_, *b* is the block index and its top left corner coordinates are (*X*_*b*_*, Y*_*b*_). The colour of each block is given by the random variable ω(*b, t*) which is taken from a Bernoulli distribution of values -1 (colour black) and 1 (colour white) with equal probability 0.5 for each block *b* at each time stamp *t*. Then, each image is randomly shifted horizontally (shift *ε*_*x*_(*t*)) and vertically (shift *ε*_*y*_(*t*)). The shifts *ε*_*x*_(*t*) and *ε*_*y*_(*t*) are random variables taking *S* possible values with a probability *1/S*. In our case, starting from images of 664 × 664 pixels (with 1 px = 4 μm), we defined the stimulus by adding 17 × 17 blocks (*B* = 289), i.e., blocks of size *P* = 40 pixels (160 μm). Choosing *S* = 4, at each time step we apply random shifts proportional to *P/S* to all blocks, so that *ε*_*x*_(*t*) and *ε*_*y*_(*t*) belong to {0*40/4 px, 1*40/4 px, 2*40/4 px, 3*40/4 px) (i.e., {0 μm, 40 μm, 80 μm, 120 μm}). As shown in Pamplona *et al*.^35^, this form of stimulus considerably improves the spatial and temporal resolution of the STA. The Michelson contrast was 0.7 with the same mean luminance stated before and SWN images were presented for 33 ms each (30 Hz, ~45 min, ~15 min for adult retinas). The spike triggered average (STA)^52^ was calculated by computing the average stimulus 500 ms (corresponding to 15 checkerboard frames) before a spike occurred. At the time point of the positive or negative peak maximum of the average temporal STA, a two-dimensional Gaussian was fitted to the corresponding spatial profile frame and an ellipse was drawn around the center with 1 SD of the Gaussian fit. The RF diameter was defined as the diameter of a circle with the same area as the ellipse (2*radius = 2 SD). We also measured the eccentricity of the fitted ellipse to see how out of round’ an RF is. The eccentricity *e* is given by:


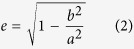


where *a* is the length of the semi-major axis (half of the longest diameter of an ellipse) and *b* is the length of the semi-minor axis (half of the shortest diameter of an ellipse). The eccentricity changes from 0 to 1, where the eccentricity of a circle is zero.

To measure STA strength at the time of the positive or negative peak maximum of the average temporal STA, we computed the z-score of STA peak amplitudes; that is for a given cell the mean of STA amplitudes was subtracted from the peak amplitude and divided by their standard deviation.

## Additional Information

**How to cite this article**: Hilgen, G. *et al*. Pan-retinal characterisation of Light Responses from Ganglion Cells in the Developing Mouse Retina. *Sci. Rep.* **7**, 42330; doi: 10.1038/srep42330 (2017).

**Publisher's note:** Springer Nature remains neutral with regard to jurisdictional claims in published maps and institutional affiliations.

## Supplementary Material

Supplementary Information

## Figures and Tables

**Table 1:** Numbers (n) of dorsal, ventral and OD ON, OFF and ON-OFF RGC response types for the different age groups used for Figs 2, 3, 4 and 5.

RGC response type		P13 (4 retinas)	P16/P17 (4 retinas)	P19 (5 retinas)	P29/P38 (4 retinas)
ON	Dorsal	1,033	392	915	1246
	Ventral	280	764	976	557
	OD	533	662	901	626
	**ON Total**	**1,846**	**1,818**	**2,792**	**2,429**
OFF	Dorsal	351	274	641	949
	Ventral	210	444	585	322
	OD	301	474	579	565
	**OFF Total**	**862**	**1,192**	**1,805**	**1,836**
ON OFF	Dorsal	706	310	822	655
	Ventral	262	527	761	295
	OD	507	557	914	596
	**ON-OFF Total**	**1,475**	**1,394**	**2,497**	**1,546**
All	**All Total**	**4,183**	**4,404**	**7,094**	**5,811**
