# Supplementary material for: Pan-retinal characterisation of Light Responses from Ganglion Cells in the Developing Mouse Retina
Source: Sci Rep. 2017 Feb 10;7:42330. doi: 10.1038/srep42330 (PMC5301206; doi:10.1038/srep42330)
Supplement: Supplementary Information [file srep42330-s1.pdf]

-Supplementary Information-

Pan-retinal characterisation of Light Responses from Ganglion Cells in the Developing Mouse Retina

Gerrit Hilgen[1*], Sahar Pirmoradian[2*], Daniela Pamplona[3], Pierre Kornprobst[3], Bruno Cessac[3,] Matthias H. Hennig[2], Evelyne Sernagor[1]

[1] Institute of Neuroscience, Newcastle University, Newcastle upon Tyne NE2 4HH, UK

[2] Institute for Adaptive and Neural Computation, University of Edinburgh EH8 9AB, Edinburgh, UK

[3] Inria, Neuromathcomp Team, 06902 Sophia Antipolis, France

* equal contributions

Supplemental Figure S1

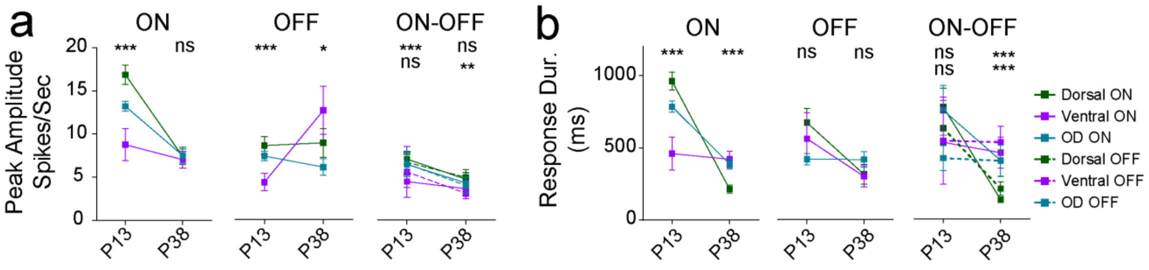

Quantification for the P13 and P38 example retinas shown in Figure 1. **a:** Peak amplitudes (*A1, A2*) for dorsal (green), ventral (purple) and OD (turquoise) ON, OFF and ON-OFF RGC responses. **b:** Response Durations (*RD1, RD2*) for dorsal (green), ventral (purple) and OD (turquoise) ON, OFF and ON-OFF RGC responses. The light on- and offset values of the ON-OFF responses were calculated as ON (solid line) and OFF (dotted line), respectively. Mean values with 95% confidence interval. Significance asterisks are only displayed for dorsal and ventral comparison: * = $p < 0.05$; ** = $p < 0.01$; *** = $p < 0.001$; ns = not significant (top ON values, bottom OFF values for ON-OFF). Detailed p-values can be found in Supplemental Table S14. n = P13 ON dorsal (D, 312), ventral (V, 42) and OD (157), P13 OFF V, D and OD (109, 32, 72, respectively), P13 ON- OFF V, D and OD (293, 72, 73); P38 ON V, D and OD (373, 218, 130), P38 OFF V, D and OD (142, 103, 73), P38 ON-OFF V, D and OD (122, 60, 61).

Supplemental Figure S2

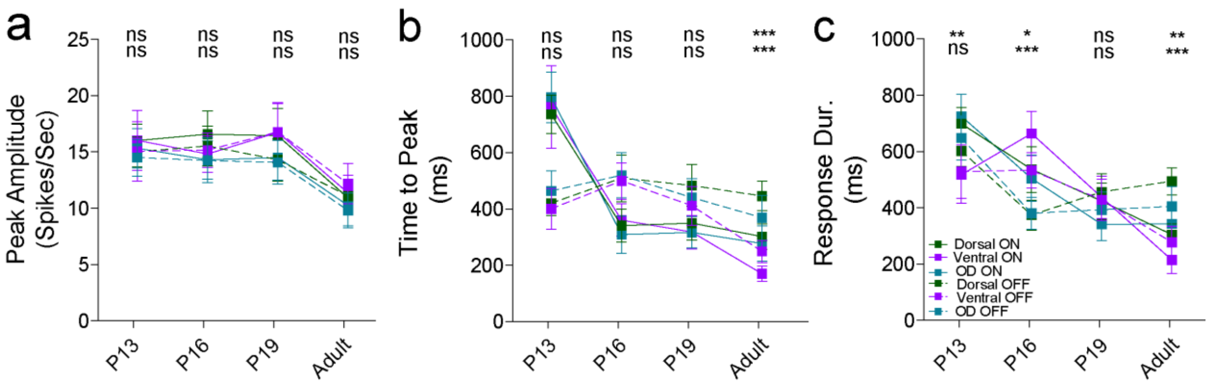

Response properties of different ON-OFF responses from P13 to adult. **a, b, c:** respectively illustrate peak amplitude (*A1*, *A2*), time to peak (*T2P1*, *T2P2*) and response duration (*RD1*, *RD2*) for dorsal (green), ventral (purple) and OD (turquoise) RGC responses. The light on- and offset values of the ON-OFF responses were calculated as ON (solid line) and OFF (dotted line), respectively. Mean values with 95% confidence interval, n: see Table 1, Significance asterisks are only displayed for dorsal and ventral comparison: * = $p < 0.05$; ** = $p < 0.01$; *** = $p < 0.001$ (top ON values, bottom OFF values). Detailed p-values can be found in Supplemental Table S9.

Supplemental Figure S3

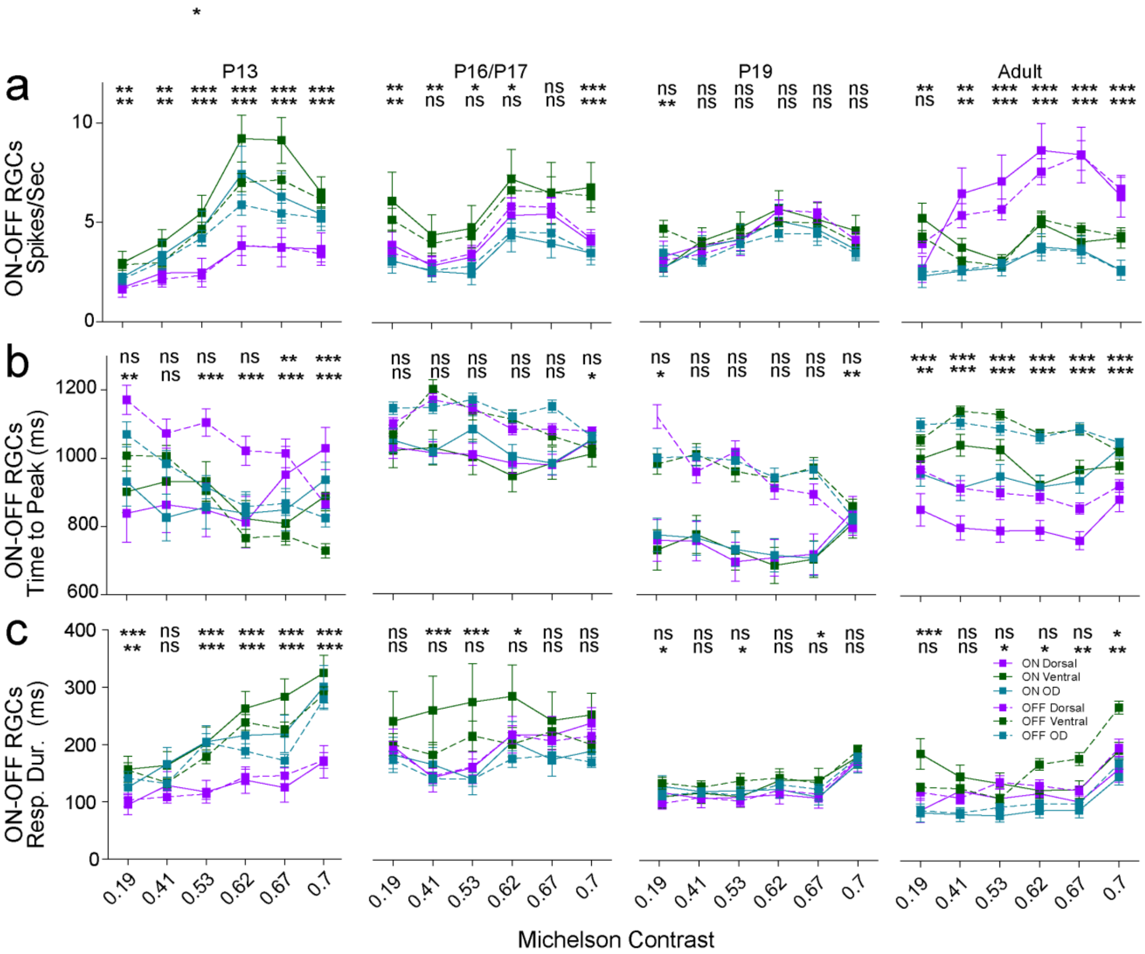

Dorsal-ventral gradient of ON-OFF response properties to different contrasts after eye-opening. **a, b, c)** Mean peak response (a), time to peak (b) and response duration (c) to different full field Michelson contrasts (0.19, 0.41, 0.53, 0.62, 0.67) for ON-OFF RGC responses for all age groups (ascending from left to right) with respect to their dorsal (green), ventral (purple) and OD (turquoise) location. For ON-OFF cells the light on- and offset values were calculated as ON (solid line) and OFF (dotted line), respectively. Mean values with 95% confidence interval, n: see Table . Significance asterisks are only displayed for dorsal and ventral comparison: * = p < 0.05; ** = p < 0.01; *** = p < 0.001 (top ON values, bottom OFF values). Detailed p-values can be found in Supplemental Table S10-12.

Supplemental Figure S4

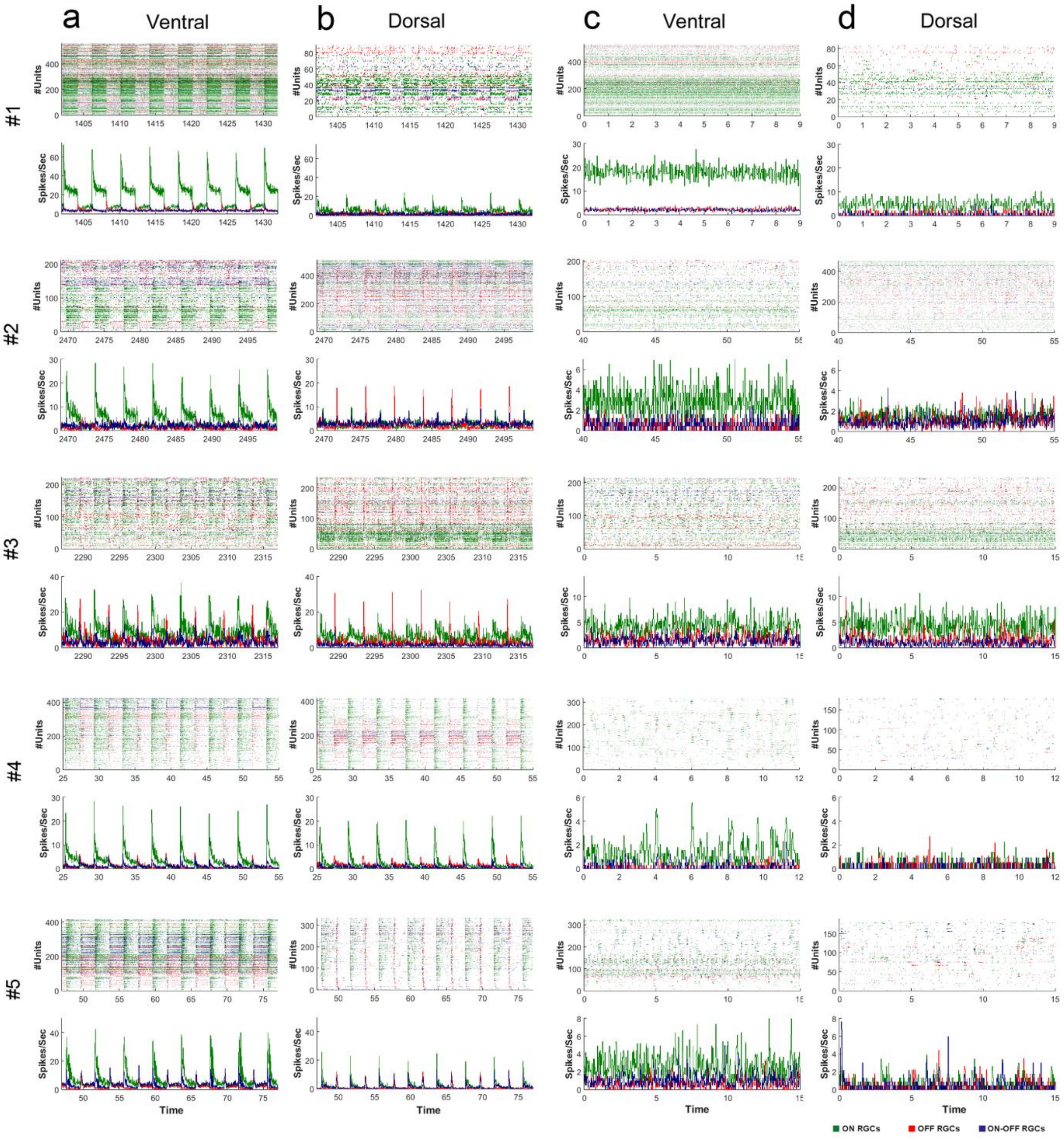

Dorsal/Ventral comparison of activity in five (rows) P19 retinas. Each dot is representing a spike within a 30 sec window from a full field stimulus (**a,b**) or within a 15 sec (8 sec for #1 and 13 sec for #4) window without any visual stimuli (**c,d**). Dots are color-coded: green = ON RGC responses, red = OFF RGC responses, dark blue = ON-OFF responses and the raster/rate plots are divided into dorsal (b,d) and ventral (a, c) RGC responses. The binned (25 ms) average response (Spikes/Sec) of all RGC responses is plotted below the raster plots. In all 5 retinas the ventral ON RGC responses are stronger (Note that ON RGC responses in #1 are more than the average stronger)

Supplemental Figure S5

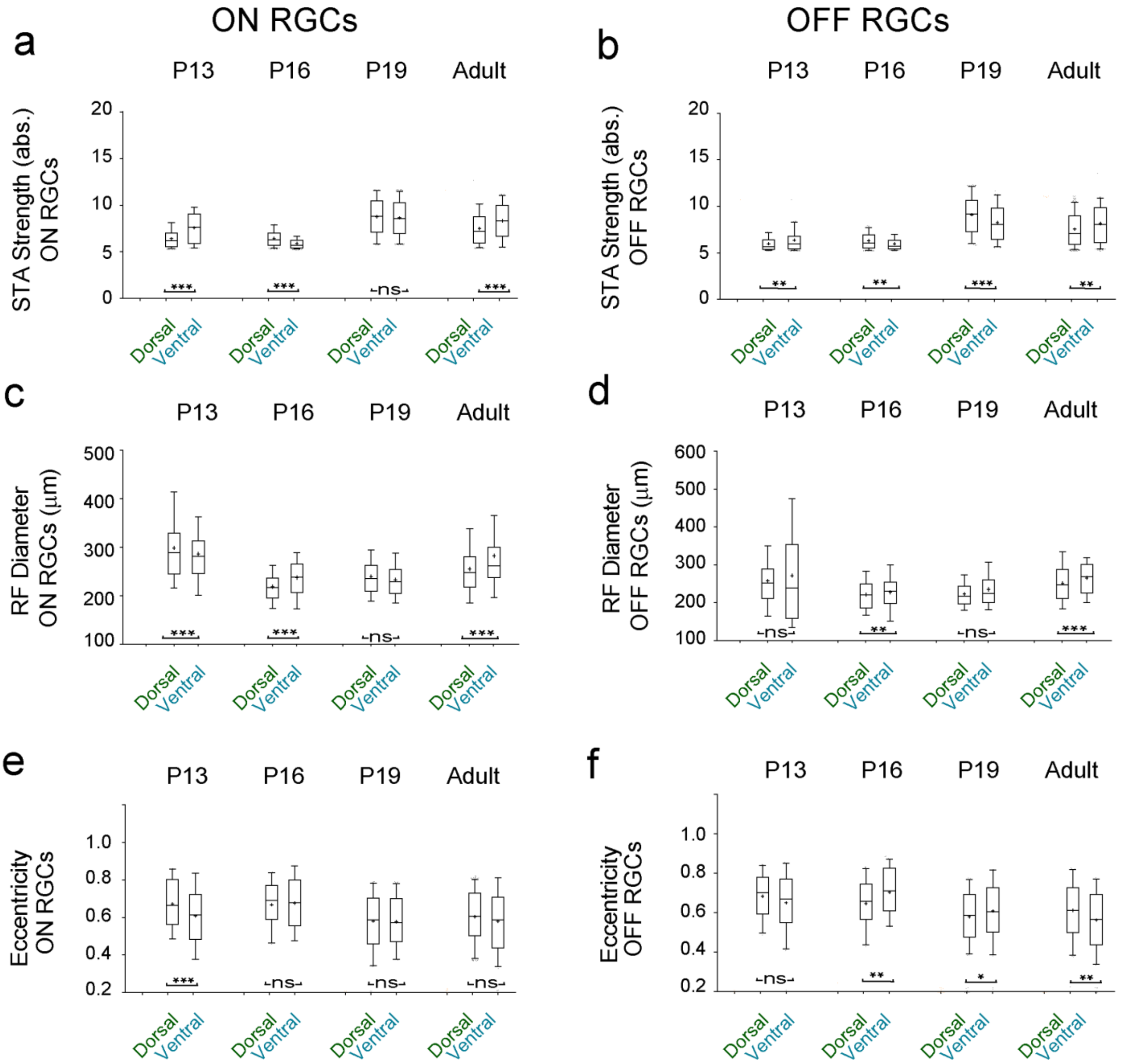

Shifted White Noise used to study the dorsal/ventral development of RGC RF central areas. **a, b:** box plots (whiskers: 10-90 percentile, mean indicated by + symbol) of RGC responses from dorsal and ventral areas for STA signal strength, RF diameters (**c, d**), and RF eccentricity (**e, f**). Significance: * = $p < 0.05$; ** = $p < 0.01$; *** = $p < 0.001$; ns = not significant. Note: For this plot we did not draw the

OD area therefore the OD RGC responses are included in either the dorsal or the ventral data. Detailed p-values can be found in Supplemental Table S13.

Supplemental Figure S6

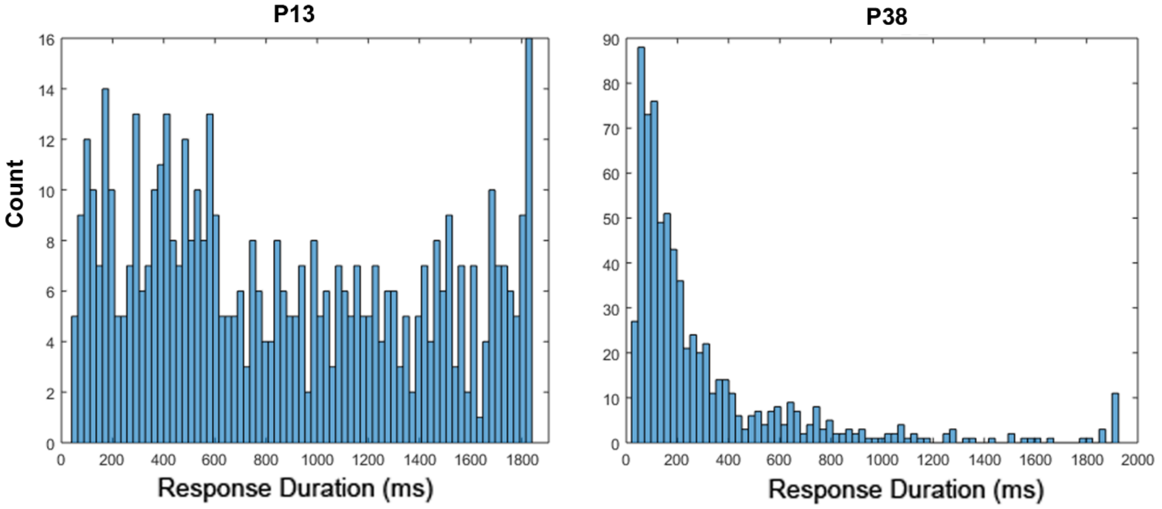

Distribution of the temporal value RD1 in a P13 (left) and an adult retina (right). For the P38, one can assume a multi-modal distribution of durations, whilst at P13, there is no clear trend in the distribution because responses are so sluggish and prolonged, and have a very high trial-to-trial variability at that developmental stage.

Supplemental Figure S7

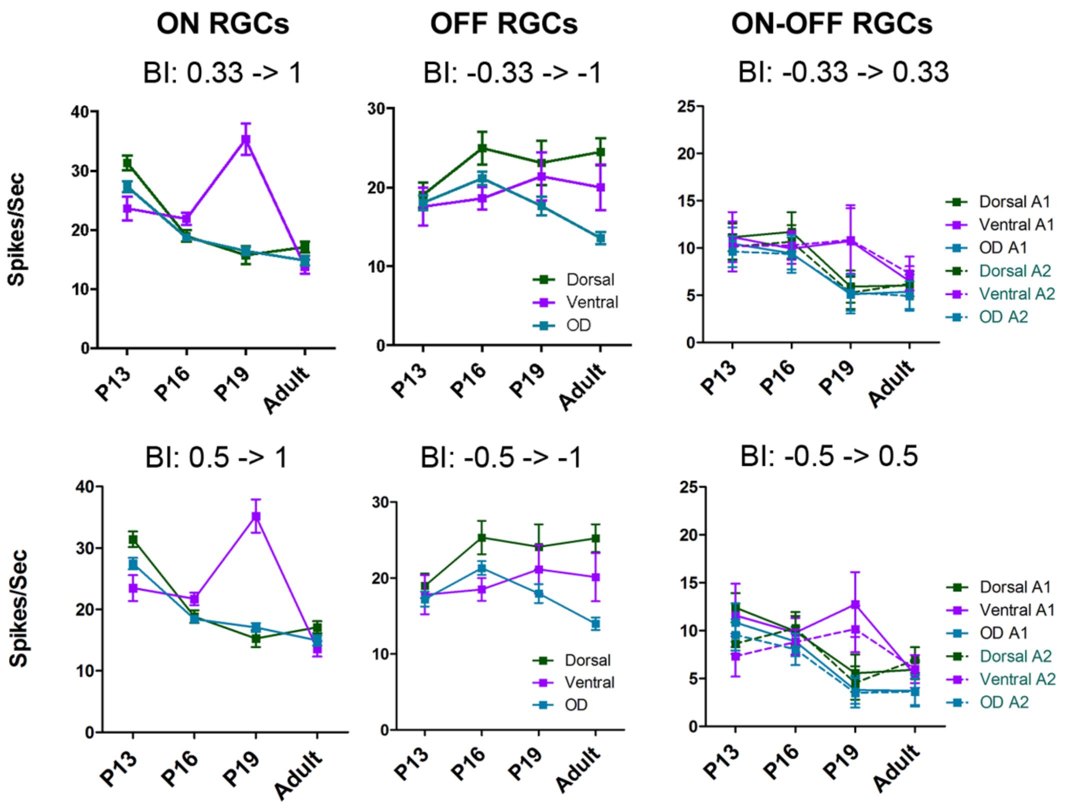

Comparison of *Bias Index* boundaries used for classification. **a, b:** The peak amplitudes (*A1*, *A2*) for dorsal (green), ventral (purple) and OD (turquoise) ON (left), OFF (middle) and ON-OFF (right) RGC responses for each age group (mean values with 95% confidence interval, n: see Table 1). We used arbitrary threshold values of 0.33 and -0.33 (a) to group our cells into ON, OFF and ON-OFF RGC responses, as this divides the entire range evenly. This is the same plot used for Figure 2a in the manuscript. We repeated the classification using threshold values of 0.5 and -0.5 (b) and observed no obvious differences in the distribution of the cell types when compared with the original values.

Supplemental Figure S8

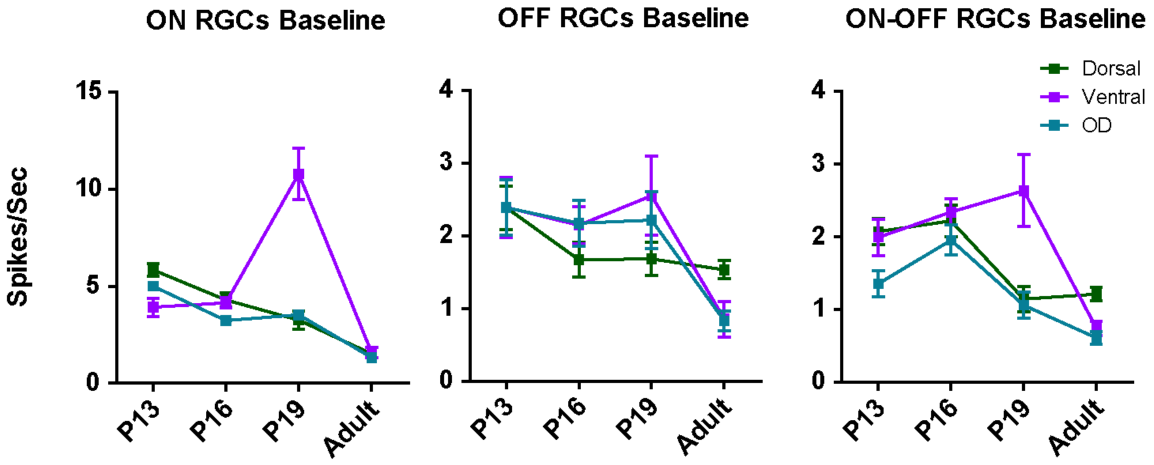

Baseline activity in ON, OFF and OFF RGC responses at different development stages. Baseline activity for dorsal (green), ventral (purple) and OD (turquoise) ON (left), OFF (middle) and ON-OFF (right) responses for each age group (mean values with 95% confidence interval, n: see Table 1). Baseline activity decreases with development except at P19 where all ventral RGC responses exhibit a significant increase in activity, most extreme for ON RGC responses. This is in line with our maximum firing rate during light responses.

Supplemental Table S9

ON Peak (A1)			ON Time to Peak (T2P1)		
**Age**	**Significant?**	**P value**	**Age**	**Significant?**	**P value**
P13	***	4,367E-09	P13	***	1,272E-06
P16	***	3,295E-04	P16		8,968E-01
P19	***	3,727E-08	P19		4,548E-01
Adult	***	5,773E-05	Adult	*	2,242E-02
**OFF Peak (A2)**			**OFF Time to Peak (T2P2)**		
P13		3,453E-01	P13		6,590E-02
P16	***	3,144E-07	P16	***	2,350E-05
P19		1,797E-01	P19		9,919E-01
Adult	**	1,621E-02	Adult		2,349E-01
**ON-OFF Peak (A1)**			**ON-OFF Time to Peak (T1P2)**		
P13		9,871E-01	P13		7,414E-01
P16		1,779E-01	P16		6,535E-01
P19		8,697E-01	P19		4,699E-01
Adult		6,625E-01	Adult	***	1,231E-04
**ON-OFF Peak (A2)**			**ON-OFF Time to Peak (T2P2)**		
P13		9,768E-01	P13		6,809E-01
P16		7,536E-01	P16		8,556E-01
P19		1,249E-01	P19		1,670E-01
Adult		3,426E-01	Adult	***	1,702E-06

ON Response Dur. (RD1)		
	**Significant?**	**P value**
P13	***	6,620E-23
P16		1,912E-01
P19	***	6,170E-11
Adult	*	3,871E-02

OFF Response Dur. (RD2)		
P13	***	4,997E-04
P16	***	2,849E-14
P19	*	3,192E-02
Adult		1,135E-01

ON-OFF Response Dur. (RD1)		
P13	**	5,125E-03
P16	*	3,325E-02
P19		7,914E-01
Adult	**	5,946E-03

ON-OFF Response Dur. (RD2)		
P13		2,362E-01
P16	***	4,546E-04
P19		5,772E-01
Adult	***	1,175E-07

Supplemental Table S10

P13 ALL Peak (A1+A2)			P13 ON Peak (A1)		
**Contrast**	**Significant?**	**P value**	**Contrast**	**Significant?**	**P value**
0,19	***	3,634E-23	0,19	***	5,858E-07
0,41	***	1,198E-22	0,41	***	1,173E-08
0,53	***	4,732E-19	0,53	***	8,179E-08
0,62	***	1,883E-32	0,62	***	1,876E-15
0,67	***	2,040E-37	0,67	***	9,977E-17
0,7	***	4,687E-31	0,70	***	5,527E-14
**P16 ALL Peak (A1+A2)**			**P16 ON Peak (A1)**		
0,19	***	4,078E-07	0,19		3,982E-01
0,41	***	6,393E-20	0,41	**	1,718E-03
0,53	***	5,346E-21	0,53	**	5,874E-03
0,62	***	1,363E-10	0,62		8,233E-01
0,67	***	2,545E-09	0,67		7,007E-01
0,7	***	3,356E-09	0,70		4,681E-01
**P19 ALL Peak (A1+A2)**			**P19 ON Peak (A1)**		
0,19	*	2,430E-02	0,19		1,032E-01
0,41	**	2,469E-03	0,41	*	3,493E-02
0,53	**	2,986E-03	0,53	*	4,654E-02
0,62	**	6,819E-03	0,62	***	9,479E-05
0,67	**	9,364E-03	0,67	***	2,749E-04
0,7	*	2,588E-02	0,7	**	1,390E-03
**Adult ALL Peak (A1+A2)**			**Adult ON Peak (A1)**		
0,19	***	2,227E-18	0,19		2,496E-01
0,41	**	3,398E-03	0,41	***	1,054E-14
0,53	***	1,820E-19	0,53	***	6,944E-19
0,62		2,860E-01	0,62		8,894E-01
0,67		9,843E-01	0,67		7,260E-01
0,7	***	1,924E-04	0,7	***	1,404E-04

P13 OFF Peak (A2)			P13 ON-OFF Peak (A1)		
**Contrast**	**Significant?**	**P value**	**Contrast**	**Significant?**	**P value**
0,19	**	6,738E-03	0,19	**	3,937E-03
0,41	***	1,062E-05	0,41	**	4,541E-03
0,53	***	3,945E-05	0,53	***	4,499E-06
0,62	**	1,217E-03	0,62	***	2,202E-08
0,67	***	9,399E-04	0,67	***	1,286E-08
0,7	**	1,022E-03	0,7	***	1,113E-05
**P16 OFF Peak (A2)**			**P16 ON-OFF Peak (A1)**		
0,19	***	3,337E-09	0,19	**	1,770E-03
0,41	***	6,266E-13	0,41	**	3,313E-03
0,53	***	5,945E-13	0,53	*	1,801E-02
0,62	***	4,934E-12	0,62	*	3,071E-02
0,67	***	2,505E-13	0,67		2,223E-01
0,7	***	5,335E-11	0,7	***	1,442E-05
**P19 OFF Peak (A2)**			**P19 ON-OFF Peak (A1)**		

0,19	**	8,036E-03	0,19		1,740E-01
0,41	*	2,462E-02	0,41		6,615E-01
0,53		6,453E-02	0,53		2,489E-01
0,62		1,141E-01	0,62		8,634E-01
0,67		1,514E-01	0,67		9,684E-01
0,7		3,156E-01	0,7		3,821E-01
**Adult OFF Peak (A2)**			**Adult ON-OFF Peak (A1)**		
0,19	**	1,403E-03	0,19	**	2,703E-03
0,41	***	2,171E-06	0,41	***	1,887E-06
0,53	***	1,173E-07	0,53	***	6,130E-15
0,62		2,055E-01	0,62	***	6,477E-08
0,67		6,305E-01	0,67	***	1,476E-12
0,7	***	8,600E-04	0,7	***	1,574E-04

**P13 ON-OFF Peak (A2)**		
**Contrast**	**Significant?**	**P value**
0,19	**	3,647E-03
0,41	**	4,570E-02
0,53	***	1,066E-05
0,62	***	2,406E-05
0,67	***	9,851E-06
0,7	***	5,857E-06
**P16 ON-OFF Peak (A2)**		
0,19	**	5,143E-03
0,41		9,690E-02
0,53		1,792E-01
0,62		3,506E-01
0,67		4,066E-01
0,7	***	5,191E-04
**P19 ON-OFF Peak (A2)**		
0,19	**	2,558E-03
0,41		3,686E-01
0,53		5,175E-01
0,62		3,708E-01
0,67		4,217E-01
0,7		8,040E-01
**Adult ON-OFF Peak (A2)**		
0,19		5,657E-01
0,41	***	3,702E-07
0,53	***	7,214E-12
0,62	***	3,162E-04
0,67	***	4,250E-08
0,7	***	1,096E-04

Supplemental Table S11

P13 ALL Peak (A1+A2)			P13 ON Peak (A1)		
**Contrast**	**Significant?**	**P value**	**Contrast**	**Significant?**	**P value**
0,19	***	1,187E-05	0,19	*	1,621E-02
0,41	***	5,378E-04	0,41	*	4,533E-02
0,53	***	1,564E-04	0,53	***	2,893E-04
0,62	***	2,555E-06	0,62	***	6,180E-05
0,67	***	3,834E-14	0,67	***	1,636E-06
0,7	***	4,089E-17	0,70	***	4,700E-10
**P16 ALL Peak (A1+A2)**			**P16 ON Peak (A1)**		
0,19		1,185E-01	0,19		9,224E-01
0,41	**	4,702E-03	0,41		1,691E-01
0,53	***	5,531E-07	0,53	*	3,638E-02
0,62	***	1,293E-05	0,62	*	3,747E-02
0,67	**	1,547E-03	0,67		5,659E-01
0,7	***	2,190E-06	0,70	**	3,147E-03
**P19 ALL Peak (A1+A2)**			**P19 ON Peak (A1)**		
0,19	*	4,216E-02	0,19		6,178E-01
0,41		9,110E-02	0,41	***	6,012E-04
0,53		4,169E-01	0,53	**	8,788E-03
0,62		3,835E-01	0,62		9,789E-01
0,67		4,088E-01	0,67		3,707E-01
0,7	**	8,683E-03	0,7		3,459E-01
**Adult ALL Peak (A1+A2)**			**Adult ON Peak (A1)**		
0,19	***	1,022E-12	0,19	***	4,622E-15
0,41	***	0,000E+00	0,41	***	0,000E+00
0,53	***	0,000E+00	0,53	***	1,661E-32
0,62	***	0,000E+00	0,62	**	5,497E-03
0,67	***	0,000E+00	0,67	***	2,547E-04
0,7	***	1,424E-08	0,7	*	3,799E-02

P13 OFF Peak (A2)			P13 ON-OFF Peak (A1)		
**Contrast**	**Significant?**	**P value**	**Contrast**	**Significant?**	**P value**
0,19		1,016E-01	0,19		2,348E-01
0,41		2,203E-01	0,41		1,797E-01
0,53	*	2,209E-02	0,53		9,394E-02
0,62		1,055E-01	0,62		8,310E-01
0,67	***	2,146E-06	0,67	**	3,264E-03
0,7	*	1,296E-02	0,7	***	3,110E-04
**P16 OFF Peak (A2)**			**P16 ON-OFF Peak (A1)**		
0,19	***	7,134E-06	0,19		7,686E-01
0,41	***	1,307E-07	0,41		6,307E-01
0,53	***	4,126E-09	0,53		8,109E-01
0,62	***	7,491E-06	0,62		1,830E-01
0,67	***	6,933E-06	0,67		8,842E-01
0,7	***	1,015E-05	0,7		7,535E-02
**P19 OFF Peak (A2)**			**P19 ON-OFF Peak (A1)**		

0,19		2,300E-01	0,19		5,209E-01
0,41	*	4,593E-02	0,41		6,352E-01
0,53		2,527E-01	0,53		4,359E-01
0,62		8,452E-01	0,62		5,529E-01
0,67		5,353E-01	0,67		7,249E-01
0,7	**	7,865E-03	0,7		2,482E-01
**Adult OFF Peak (A2)**			**Adult ON-OFF Peak (A1)**		
0,19	***	5,97E-07	0,19	***	9,795E-06
0,41	***	1,922E-26	0,41	***	5,101E-16
0,53	***	3,610E-30	0,53	***	4,788E-18
0,62	**	5,714E-03	0,62	***	1,411E-08
0,67	***	7,433E-06	0,67	***	4,758E-16
0,7		1,358E-01	0,7	***	8,521E-06

**P13 ON-OFF Peak (A2)**		
**Contrast**	**Significant?**	**P value**
0,19	**	2,538E-03
0,41		2,189E-01
0,53	***	1,361E-04
0,62	***	1,532E-07
0,67	***	1,486E-06
0,7	***	2,819E-04
**P16 ON-OFF Peak (A2)**		
0,19		3,223E-01
0,41		3,449E-01
0,53		7,866E-01
0,62		3,438E-01
0,67		5,194E-01
0,7	*	2,045E-02
**P19 ON-OFF Peak (A2)**		
0,19	**	2,323E-03
0,41		2,616E-01
0,53		1,908E-01
0,62		5,156E-01
0,67		7,432E-02
0,7	*	3,967E-02
**Adult ON-OFF Peak (A2)**		
0,19	*	1,483E-02
0,41	***	1,383E-12
0,53	***	1,251E-15
0,62	***	6,165E-12
0,67	***	4,869E-17
0,7	***	4,714E-06

Supplemental Table S12

P13 ALL Peak (A1+A2)			P13 ON Peak (A1)		
**Contrast**	**Significant?**	**P value**	**Contrast**	**Significant?**	**P value**
0,19	***	3,044E-16	0,19	*	3,017E-02
0,41	***	2,449E-13	0,41	***	2,546E-07
0,53	***	8,745E-19	0,53	***	2,148E-09
0,62	***	6,996E-29	0,62	***	2,080E-15
0,67	***	5,992E-33	0,67	***	3,515E-21
0,7	***	0,000E+00	0,70	***	6,046E-30
**P16 ALL Peak (A1+A2)**			**P16 ON Peak (A1)**		
0,19	*	2,510E-02	0,19		9,397E-01
0,41	**	6,211E-03	0,41	***	3,397E-04
0,53	***	4,332E-05	0,53	***	1,840E-05
0,62	***	2,751E-04	0,62	***	5,802E-09
0,67	***	6,054E-06	0,67	***	4,910E-09
0,7		3,662E-01	0,70	*	1,422E-02
**P19 ALL Peak (A1+A2)**			**P19 ON Peak (A1)**		
0,19		2,275E-01	0,19		6,869E-02
0,41		4,210E-01	0,41		3,865E-01
0,53		7,902E-01	0,53	**	1,967E-03
0,62	**	8,811E-03	0,62	***	6,790E-05
0,67	***	8,218E-04	0,67	***	7,341E-08
0,7	***	5,467E-04	0,7	***	3,805E-06
**Adult ALL Peak (A1+A2)**			**Adult ON Peak (A1)**		
0,19	***	1,849E-14	0,19	***	1,607E-06
0,41	***	5,383E-11	0,41		2,715E-01
0,53	***	3,238E-07	0,53		4,011E-01
0,62	***	2,347E-16	0,62	***	4,826E-07
0,67	***	5,665E-19	0,67	***	1,408E-09
0,7		5,786E-02	0,7		4,981E-01

P13 OFF Peak (A2)			P13 ON-OFF Peak (A1)		
**Contrast**	**Significant?**	**P value**	**Contrast**	**Significant?**	**P value**
0,19		5,217E-02	0,19	***	2,313E-04
0,41		7,149E-01	0,41		6,690E-02
0,53	***	2,800E-04	0,53	***	2,813E-05
0,62	*	1,098E-02	0,62	***	2,474E-07
0,67	*	1,583E-02	0,67	***	5,701E-10
0,7	***	2,506E-06	0,7	***	5,989E-10
**P16 OFF Peak (A2)**			**P16 ON-OFF Peak (A1)**		
0,19		7,401E-02	0,19		1,492E-01
0,41		2,316E-01	0,41	***	3,739E-05
0,53		3,582E-01	0,53	***	2,167E-04
0,62	**	2,257E-03	0,62	*	3,240E-02
0,67		7,393E-02	0,67		4,108E-01
0,7	***	4,888E-08	0,7		5,637E-01
**P19 OFF Peak (A2)**			**P19 ON-OFF Peak (A1)**		

0,19		5,933E-02	0,19		6,087E-01
0,41		1,367E-01	0,41		4,319E-01
0,53	**	8,842E-03	0,53		9,216E-01
0,62		6,333E-01	0,62		7,140E-02
0,67		8,105E-01	0,67	*	2,875E-02
0,7		1,409E-01	0,7		6,810E-01
**Adult OFF Peak (A2)**			**Adult ON-OFF Peak (A1)**		
0,19	***	8,09E-08	0,19	***	7,790E-04
0,41	*	1,684E-02	0,41		1,966E-01
0,53	*	3,477E-02	0,53		1,018E-01
0,62	**	1,497E-03	0,62		6,489E-01
0,67	***	5,895E-04	0,67		1,074E-01
0,7	*	1,881E-02	0,7	*	2,906E-02

P13 ON-OFF Peak (A2)

Contrast	Significant?	P value
0,19	**	9,117E-03
0,41		8,947E-02
0,53	***	2,601E-04
0,62	***	2,204E-05
0,67	***	1,066E-04
0,7	***	4,976E-07
**P16 ON-OFF Peak (A2)**		
0,19		7,391E-01
0,41		1,542E-01
0,53		5,317E-02
0,62		5,523E-01
0,67		5,646E-01
0,7		4,818E-01
**P19 ON-OFF Peak (A2)**		
0,19	*	1,425E-02
0,41		2,199E-01
0,53	*	2,392E-02
0,62		1,493E-01
0,67		1,957E-01
0,7		3,092E-01
**Adult ON-OFF Peak (A2)**		
0,19		5,887E-01
0,41		2,901E-01
0,53	*	3,704E-02
0,62	*	3,591E-02
0,67	**	4,118E-03
0,7	**	1,222E-03

Supplemental Table S13

STA Strength ON			STA Strength OFF		
**Pairs**	**Significant?**	**P value**	**Pairs**	**Significant?**	**P value**
P13 all vs P16 all	***	6,730E-27	P13 all vs P16 all		3,993E-01
P13 all vs P19 all	***	4,058E-37	P13 all vs P19 all	***	0,000E+00
P13 all vs Adult all	***	1,005E-04	P13 all vs Adult all	***	1,678E-31
P16 all vs P19 all	***	0,000E+00	P16 all vs P19 all	***	0,000E+00
P16 all vs Adult all	***	3,533E-45	P16 all vs Adult all	***	1,844E-29
P19 all vs Adult all	***	1,053E-24	P19 all vs Adult all	***	8,896E-17
P13 dorsal vs P13 ventral	***	4,294E-12	P13 dorsal vs P13 ventral	**	4,138E-03
P16 dorsal vs P16 ventral	***	5,353E-10	P16 dorsal vs P16 ventral	**	5,337E-03
P19 dorsal vs P19 ventral		3,842E-01	P19 dorsal vs P19 ventral	***	2,227E-07
Adult dorsal vs Adult ventral	***	1,757E-06	Adult dorsal vs Adult ventral	**	1,406E-03

RF Diameter ON			RF Diameter OFF		
**Pairs**	**Significant?**	**P value**	**Pairs**	**Significant?**	**P value**
P13 all vs P16 all	***	9,189E-11	P13 all vs P16 all		3,989E-01
P13 all vs P19 all	***	7,486E-32	P13 all vs P19 all	**	1,527E-03
P13 all vs Adult all	***	5,011E-36	P13 all vs Adult all	***	3,556E-06
P16 all vs P19 all	***	2,232E-11	P16 all vs P19 all	***	2,414E-04
P16 all vs Adult all	***	6,822E-18	P16 all vs Adult all	***	4,461E-09
P19 all vs Adult all	**	4,404E-03	P19 all vs Adult all	*	1,091E-02
P13 dorsal vs P13 ventral	***	2,312E-10	P13 dorsal vs P13 ventral		7,517E-01
P16 dorsal vs P16 ventral	***	1,691E-11	P16 dorsal vs P16 ventral	**	7,429E-03
P19 dorsal vs P19 ventral		4,094E-01	P19 dorsal vs P19 ventral		8,829E-01
Adult dorsal vs Adult ventral	***	2,106E-15	Adult dorsal vs Adult ventral	***	3,507E-22

Eccentricity ON			Eccentricity OFF		
**Pairs**	**Significant?**	**P value**	**Pairs**	**Significant?**	**P value**
P13 all vs P16 all	***	1,630E-05	P13 all vs P16 all		5,538E-01
P13 all vs P19 all	***	6,790E-07	P13 all vs P19 all	***	2,612E-11
P13 all vs Adult all	**	7,662E-03	P13 all vs Adult all	***	1,631E-08
P16 all vs P19 all	***	3,689E-23	P16 all vs P19 all	***	1,414E-11
P16 all vs Adult all	***	1,483E-13	P16 all vs Adult all	***	6,267E-09
P19 all vs Adult all	*	1,409E-02	P19 all vs Adult all		6,528E-01
P13 dorsal vs P13 ventral	***	2,172E-04	P13 dorsal vs P13 ventral		7,222E-02
P16 dorsal vs P16 ventral		5,801E-01	P16 dorsal vs P16 ventral	**	3,930E-03
P19 dorsal vs P19 ventral		7,595E-01	P19 dorsal vs P19 ventral	*	1,107E-02
Adult dorsal vs Adult ventral		9,875E-02	Adult dorsal vs Adult ventral	**	1,624E-03

Supplemental Table S14

ON Peak (A1)			ON RD (A1)		
**Age**	**Significant?**	**P value**	**Age**	**Significant?**	**P value**
P13	***	6,839E-07	P13	***	3,270E-08
P38		6,215E-01	P38	***	3,626E-12
**OFF Peak (A2)**			**OFF RD (A2)**		
**Age**			**Age**		
P13	***	4,726E-05	P13		2,833E-01
P38	*	1,617E-02	P38		7,803E-01
**ON-OFF Peak (A1)**			**ON-OFF RD (A1)**		
**Age**			**Age**		
P13	***	1,254E-04	P13		1,473E-01
P38		9,030E-02	P38	***	5,920E-14
**ON-OFF Peak (A2)**			**ON-OFF RD (A2)**		
**Age**			**Age**		
P13		4,848E-01	P13		5,658E-01
P38	**	6,070E-03	P38	***	4,165E-09
